# Examining the Antecedent Role of Movement Proficiency in Child Development: Study Protocol

**DOI:** 10.3389/fpsyg.2021.678874

**Published:** 2021-07-15

**Authors:** Catherine M. Capio, Kerry Lee, Rachel A. Jones, Rich S. W. Masters

**Affiliations:** ^1^Department of Early Childhood Education, The Education University of Hong Kong, Hong Kong, China; ^2^Health Science Department, Ateneo de Manila University, Quezon City, Philippines; ^3^School of Education, Early Start, University of Wollongong, Wollongong, NSW, Australia; ^4^Te Huataki Waiora School of Health, University of Waikato, Hamilton, New Zealand

**Keywords:** gross motor skills, executive function, social behavior, movement training, early childhood

## Abstract

**Background:** Decades of research, largely from associational studies, show that the relationships of movement proficiency with the cognitive and social aspects of development are particularly strong in early childhood. Children who move proficiently tend to have better cognitive skills and social behaviors. However, the mechanisms that underpin these relationships remain unclear and research that explores causation is necessary. This study will explore the antecedent role of movement proficiency in the cognitive and social domains of child development, by examining whether a targeted movement skills training program facilitates improvements in cognitive and social skills.

**Methods:** A group-randomized controlled trial will be conducted, implementing a fundamental movement skills training program in Hong Kong kindergartens. Participants will consist of children aged 3–5 years (*N* = 158) who will be randomly allocated by class to either a training or active control condition. The training program (10 weeks × 2 bouts) will be informed by an error-reduced approach to skills learning, which will involve careful design and manipulation of equipment and training environment to minimize practice errors. The active control condition will consist of typical movement activities implemented in the kindergartens in the context of the local curriculum guide. Outcomes will be measured using standardized tests of gross motor skills proficiency, executive functioning, and social skills. Measurements will occur at baseline, mid-training, post-training, and follow-up. Latent variable longitudinal modeling will be used to analyze changes in the outcomes, with covariates that include sex, body composition, fine motor skills, and physical activity.

**Expected Results:** The findings will subsequently be reported consistent with the Consolidated Standards of Reporting Trials (CONSORT) statement. Contributions to knowledge and understanding of child development are expected, through evidence of causal mechanisms surrounding the relationship of motor with cognitive and social development. The findings will also inform policy and practice related to early childhood development and education.

## Introduction

A child who is proficient at moving is enabled to interact with the environment in increasingly complex ways. Early on, Piaget (1963) noted that motor skills influence the number and types of opportunities for children to interact with others. From a dynamic systems perspective (Thelen and Smith 1996; Thelen, 2005), a relatively small change in motor development – enabled by such interactions – can have an escalating impact on social and cognitive functions.

Motor skills are key contributors to children’s ability to play and interact with others, therefore influencing their later social standing with peers. For instance, poor gross motor skills have been related to weakness in emotion comprehension in early childhood (Piek et al., 2008a). Longitudinal studies have also shown that motor skills at the age of five to six years predicted adaptation and social behaviors in school one year later (Bart et al., 2007); motor skills at the age of six to seven years influenced social status among peers at nine to ten years (Ommundsen et al., 2010). This could be related to the observation that social play is reduced and social reticence is heightened in children with poor motor skills (Bar-Haim and Bart, 2006). Early peer acceptance is also known to be related to adult adjustment (Bagwell et al., 1998) and later academic achievement (Wentzel and Caldwell, 1997), suggesting that there may be long-term consequences to the risks that poor motor skills in early childhood pose on psychosocial development. This highlights the importance of investing in motor skills development in early childhood to mitigate future wellbeing problems.

Gross motor skills in early childhood have also been shown to be associated with cognitive development (Veldman et al., 2019) and to predict levels of cognitive processing later in life (Murray et al., 2006; Piek et al., 2008b). From longitudinal studies, early gross motor skills have been shown to predict cognitive efficiency (Lopes et al., 2013; Haapala et al., 2014) and academic achievement in subsequent school transition (Niederer et al., 2011; Roebers et al., 2014). A review of intervention programs suggested that increasing the amount of physical and movement activities might generate improvements in executive functioning of school-aged children (Diamond and Ling, 2016). However, the mechanisms through which movement-based interventions can improve executive function have yet to be fully understood.

Executive function is an umbrella term that refers to a set of higher order cognitive processes (e.g., inhibitory control, working memory, and cognitive flexibility) that are associated with the prefrontal cortex area of the brain (Hughes and Ensor, 2011). Core components of executive function develop during early childhood, forming a critical foundation for subsequent development of cognitive processes in adulthood (Garon et al., 2008). McClelland and Cameron (2019) highlighted that both executive function and motor skills are foundational skills that should be nurtured during early childhood. The years from three to five make up an important period in the development of executive function (Garon et al., 2008) and would presumably be the ideal age to examine whether they can be enhanced alongside motor enrichment.

The relationship between motor skills and other developmental domains appears to be strongest during the early years of life (Libertus and Hauf, 2017). This suggests that a robust foundation of movement proficiency in early childhood may be a critical antecedent to the development of other domains. However, the state of evidence, as discussed above, has been largely drawn from associational studies; there is a knowledge gap that limits our understanding of the mechanisms through which the movement proficiency influences the other domains of child development. Experimental designs are needed to reveal the underlying mechanisms or processes that drive development and establish causal relationships (Getchell et al., 2020).

Previous experimental studies that examined the impact of movement-focused programs on other developmental domains have tended to focus on primary school-aged children. For instance, a group-randomized physical education intervention involved children aged 5 to 10 years (Pesce et al., 2016). Improved motor coordination was accompanied by gains in executive functioning and attention, but only when children engaged in physical activity on their own. In contrast, an after-school program for early primary school children that trained fundamental movement skills did not yield significant benefits in cognitive functions (Lee et al., 2020). It was noted that the sample size of this other study was relatively small, and cognitive functioning was measured with a parent-proxy report.

There have been relatively limited intervention studies in pre-primary school-aged children. One such study in South Africa found that a community-based movement program was beneficial for both gross motor skills and cognitive functioning among children aged three to six years from disadvantaged communities (Draper et al., 2012). The researchers speculated that improvements in both cognitive functioning and gross motor skills could simply be attributed to the general stimulation afforded by participating in the program, considering the general lack of space and resources for activities prior to implementation of the program. The extent to which movement training contributed to improvements in cognitive functioning remained unclear, and the mechanisms that facilitated the improved motor skills and cognitive functioning were unexplored. It is therefore necessary that experimental studies that implement movement skills training are conducted in which mechanisms and interacting factors can be examined (Getchell et al., 2020).

### Movement Skills Training of Children

Skills, such as locomotor and object manipulation, are not acquired innately as children grow up (Clark, 2005). Instead, planned programs are needed to facilitate the skills acquisition during early childhood (Logan et al., 2012). How should movement skills be trained in early childhood? Masters et al. (2013) suggested that the stage learning models of motor skill acquisition (Fitts and Posner, 1967) seem inappropriate during early childhood. Stage models suggest that a learner goes through an initial cognitive stage when learning motor skills during which methods to perform movements successfully are formulated, and the validity of perceived feedback is judged. In this stage, there is heavy reliance on cognitive resources and language ability to process and formulate rules and methods for movement performance; cognitive and verbal engagement is reduced later after extensive practice. Considering that cognitive resources and language skills are not fully developed in early childhood, it is unlikely that initiating a cognitive stage of skill learning as described above would be ideal for children at this stage.

In training motor skills during early childhood, it is therefore necessary to consider that skills may be best acquired through less engagement of verbal processes (e.g., instructions and rules). Masters and colleagues have developed this approach under the label of implicit motor learning (Masters, 1992; see Masters et al., 2019, for a recent review). Neurocognitive research has shown that young children tend to rely more on visual codes and less on verbal labels (Alloway et al., 2006) and rely less on executive resources when performing dual tasks compared to older children (Ang and Lee, 2010). Grounded on this evidence, an approach to training motor skills wherein practice errors are minimized may be suitable during early childhood.

When learners commit practice errors, they seek alternative movement solutions, form rules to support successful performance, and consequently draw upon their working memory resources (Maxwell et al., 2001). On the contrary, when practice errors are minimized, motor skills are learnt with less reliance on cognitive resources, which is suitable for children whose cognitive functions are still developing (Capio et al., 2012). This is the basis of the error-reduced approach, which has been shown to improve gross motor skills proficiency of children without dependence on short-term memory capacity (Capio and Eguia, 2020).

In earlier work, the error-reduced approach was tested specifically in the training of overhand throwing by children (Capio et al., 2013a). Practice errors were limited in the initial stage of learning by incrementally manipulating task difficulty (i.e., throwing at targets that were large enough to ensure high success rates). Movement performance was found to improve; children displayed the ability to engage in a secondary cognitive task (i.e., counting backward) without it disrupting movement performance. This suggested that movement performance was not dependent on cognitive resources. The findings were replicated in children with cognitive limitations (Capio et al., 2013b), providing further evidence that this approach is relatively less reliant on cognitive resources. Interestingly, children who trained with an error-reduced approach manifested a greater increase in spontaneous throwing activity during free play. Capio et al. (2013b) suggested that greater experiences of success during practice facilitated subsequent sustained engagement and motivation; these two factors have been shown to mediate the positive effect of movement training on cognitive development (Pesce et al., 2016). As such, the error-reduced training could be an approach that not only improves movement performance but also aids cognitive development. Greater experience of success during practice has been associated with enhanced self-efficacy (Muller and Roder, 2010), which suggests that it could contribute to psychosocial development in early childhood. While converging evidence from studies involving children of different abilities (i.e., Maxwell et al., 2017; Capio et al., 2018) has indicated the value of an error-reduced approach to motor skills training, the potential effects on cognitive and social development have yet to be verified.

### Research Gap and the Present Study

It is evident that movement skills proficiency plays a crucial role in optimal cognitive and social development in early childhood. Children who have proficient motor skills tend to have positive outcomes in a number of child development domains. The evidence to date, however, has been largely drawn from studies of association, and the mechanisms underlying the apparent relationships between the motor domain and other domains of development continues to be poorly understood (Libertus and Hauf, 2017). With limited evidence from intervention studies, the antecedent role of movement proficiency in child development is an area that needs further exploration. It remains unclear whether enhanced movement proficiency can directly impact cognitive and social development in early childhood, or whether all developmental domains are similarly influenced by factors, such as general stimulation or the early childhood education environment.

From a theoretical perspective, it has been noted that motor development has been relatively neglected when trying to understand childhood behaviors (Rosenbaum, 2005; Iverson, 2010), even though movement enables exploration and expression. This may be due in part to limited evidence from robust experimental designs, which this present study aims to address. The aim of this study is to examine whether improved movement proficiency leads to benefits in the cognitive and social domains of child development. A fundamental movement skill training program, using the error-reduced learning approach, will be implemented and compared with typical kindergarten activities (i.e., active control). It is hypothesized that significant and meaningful improvements will be observed in gross motor skills proficiency from baseline to immediate and delayed time points following training; improvements will be greater among those in an error-reduced condition compared to those in the active control group. It is further hypothesized that improvements will be found in executive functioning and social competence from baseline to immediate and delayed time points following training; improvements will be greater for those who displayed larger gains in gross motor skills proficiency. Executive functions will be based on standardized tests of inhibitory control and working memory, while social competence will be based on standardized reports by teachers. Factors that could influence the main variables of interest will be accounted for.

## Materials and Methods

### Context and Design

The study will be implemented in Hong Kong, where children aged three to five years attend kindergarten classes. All local kindergartens follow the same curriculum which stipulates a learning area of physical fitness and health, inclusive of fundamental movement skills development (Curriculum Development Council, 2017). This is a two-arm (training vs. active control), group-randomized intervention study that is situated in the kindergarten setting; the intervention will be delivered in partnership with teachers.

### Participants

A purposive sampling approach will be taken with the following inclusion criteria for participants: Children (1) aged three to five years, (2) enrolled in a local kindergarten within the same territory as the research center (i.e., New Territories and Hong Kong), and (3) have no diagnosed neurodevelopmental, medical, or orthopedic condition that is contraindicated to moderate intensity physical activity or that requires special educational needs support. In previous research, a six-week gross motor skills training program led to improved motor proficiency, with effect sizes ranging from 0.28 to 0.45 (Brian and Taunton, 2018). Taking a conservative approach, it was calculated that to achieve 80% power (two-tailed alpha at 0.05), with an effect size of 0.28, and accounting for between-group and within-group interactions, a total sample of 144 is required to compare two groups (i.e., training and control) at four testing points. Accounting for 10% potential attrition rate, 79 participants per group (*N* = 158) will be recruited. The participants will be randomly allocated as a group (i.e., one intact kindergarten class) to either training or active control. Typical kindergarten classes in Hong Kong have about 20 to 30 pupils; hence, three to four classes will be allocated to each group (i.e., training and active control). To control for a cluster effect and socioeconomic status being a potential confounder, we had recruited a local kindergarten with at least eight kindergarten classes from a middle-class area.

### Procedures

**Figure 1:**
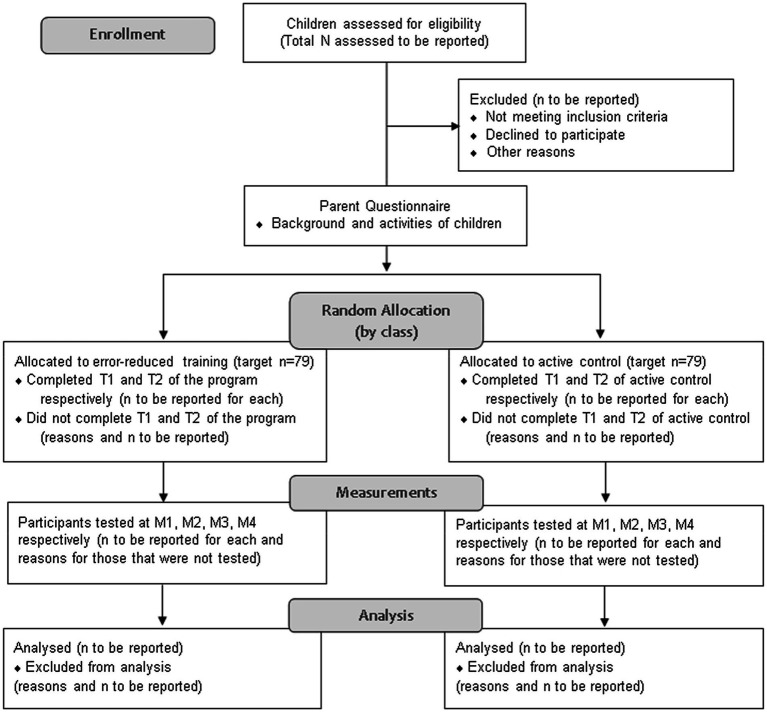
Participants’ planned progress through the study.

Random allocation will be at group level because the intervention is to be delivered in the context of kindergarten classes. Figure 1 illustrates the planned progress of participants through the study, following the flow diagram recommended by the Consolidated Standards of Reporting Trials (CONSORT; Schulz et al., 2010). Following receipt of parental informed consent, participants’ parents will be invited to complete a questionnaire ascertaining background information about the child, including typical daily activities and other learning (e.g., phonics and music) and physical (e.g., after-school sports and cycling) activities outside of the kindergarten. It is expected that parents will take about 20 to 30 min to complete the questionnaire. Measurement of covariate and outcome variables will be conducted across four time points, with the training program commencing after the first measurement (see Table 1 for schedule of implementation).

**Table 1:** Schedule of measurements and training.

Measurement/Training	M1	T1	M2	T2	M3	M4
Physical growth: height (cm) and body mass (kg)	•					
Gross motor skills proficiency	•		•		•	•
Fine motor skills	•					•
Physical activity and sedentary behavior	•					•
Executive function	•		•		•	•
Social competence	•		•		•	•
Intervention/active control		•		•		

*M1, baseline measurement; T1, term 1 training; M2, mid-training measurement; T2, term 2 training; M3, post-training measurement; and M4, follow-up measurement. Outcomes are measured across all time points; covariates are measured only at baseline and follow-up*.

Preliminary work was conducted with local kindergarten teachers, which identified a number of key design considerations for the movement training program. First, training will target fundamental movement skills comprising: object control (i.e., throwing, catching, and kicking), locomotion (i.e., running, jumping, and hopping), and stability (i.e., turning, rolling, and balancing; Ulrich, 2000; Donnelly et al., 2017). These specific skills were identified to be consistent with the expectations of the curriculum guide for kindergartens in Hong Kong (Curriculum Development Council, 2017). Second, 20-min training sessions, three times per week, were deemed feasible in the context of the typical programming of local kindergartens. Third, the program duration was balanced to be both evidence-informed and fit for the local context. Significant movement skills improvement was evident following eight weeks of error-reduced training (Capio et al., 2018) or six weeks of training delivered by early childhood teachers (Brian et al., 2017). However, it was noted that eight weeks of fundamental movement skills training did not improve cognitive functioning of early primary school children (Lee et al., 2020). Therefore, the program duration in this study will be extended to enable motor development (as opposed to skill acquisition). Working around the school terms and breaks of the local curriculum, a 10-week intervention was deemed feasible in each of the two semesters of one school year. Finally, it was determined that teachers will be trained to deliver the intervention, supported by project assistants who had been trained in error-reduced approach to fundamental movement skills training. This was deemed an important consideration because teacher-led interventions in early childhood settings have been shown to benefit children (Jones et al., 2016). Teachers’ training will consist of the knowledge base for the error-reduced learning approach, the rationale for each lesson plan, and the performance measures that would serve as the basis for progression.

Errors will be minimized by specifically manipulating equipment size and training conditions, such that the initial sessions are suitably easy to allow successful movement performance. Progressive increments in task difficulty will follow when each child in a class is able to display successful performance ≥75% of the time at a task (e.g., task completion: 6 out of 8 attempts are successful). Equipment manipulation has been shown to be feasible in field-based studies (e.g., Buszard et al., 2014), and delivering error-reduced motor skills training in class settings is feasible (Capio et al., 2015). Class-based training activities were therefore designed in partnership with local kindergarten teachers, where equipment and environment set-up, procedures, and progressions were identified (see Table 2 for a sample).

**Table 2:** Sample activity for the movement training program.

Skill	Equipment/Set-up	How	Progressions	Performance measure
Hopping	*Equipment* Circle mats Masking tape or carpet squares *Setting* Indoor or outdoor Set-up rows of three single mats and three double mats	The teacher will demonstrate hopping on one leg through the row of single mats, and jumping (two legs) through the row of double mats The children will take turns in hopping on single mats and jumping on double mats. Hopping will be practiced with both right and left legs Note that the focus is on hopping, but jumping is included for activity variation	Initial session will be hopping on the spot, while the children hold on to support (hold on to shoulders of classmates) Hopping in place without holding on to support Hop through the row of single mats in pairs (children holding hands for support). Hop through the row of single mats individually *Equipment manipulations to further increase task difficulty* Increase the number of mats in a row Increase the distance between consecutive mats	Successful hopping on the spot/in place at least 6 out of 8 trials in each bout to progress Successful hopping through a full row of mats at least 3 out of 4 rounds to progress

*Group progression will take place when each child in a class is able to achieve ≥75% successful performance*.

The active control group will participate in the typical physical activities currently being implemented in their kindergartens in the context of the local curriculum guide. Details of the activities (including the parameters, such as repetitions and bouts) completed during the study period will be documented for both the active control and the training group to ensure that activities are delivered as planned (i.e., fidelity check). The instruction approach for the active control groups will also be identified and documented (e.g., direct instruction).

### Measures

To measure immediate and sustained effects of training on the outcomes, assessments will be performed prior to training (baseline, M1), at the end of the first term (mid-training, M2), at the end of the second term (post-training, M3), and four months after the end of training (follow-up, M4). Participants’ performances in the tests for fundamental movement skills, executive function, and working memory will be recorded on videos and scored post-hoc by designated research staff who were not involved in the test administration (i.e., blinded to participants’ allocation to training or active control groups). A random sample of video recordings (i.e., 10%) will be scored by a second set of designated research staff to establish inter-rater reliability.

#### Outcome: Gross Motor Skills

The standardized Test of Gross Motor Development – 3rd Edition (TGMD-3; Ulrich, 2017, 2019) – will be used to measure gross motor skills proficiency. TGMD measures gross motor skills development of children (Ulrich, 2000) and had been widely used in research and practice (e.g., Chow and Chan, 2011; Bandeira et al., 2020). TGMD-3 is the recently updated version and had been found to have high levels of validity, internal consistency, and reliability (Webster and Ulrich, 2017).

Test of Gross Motor Development-3 (Ulrich, 2019) consists of two sub-scales: locomotor (run, gallop, skip, jump, hop, and slide) and object control skills (two-hand strike, one-hand strike, dribble, catch, kick, overhand throw, and underhand throw). For each skill, the examiner will demonstrate the skill, after which the child will perform one practice trial and two scored trials. A skill is scored based on the presence (score of 1) or absence (score of 0) of each criterion in every trial; the score for each skill is the sum of all criteria scores from two trials. Depending on the skill, performance criteria range from three to five across skills. The highest possible raw scores for the locomotor and object control sub-scales are 46 and 54, respectively (highest possible overall score is 100). Post-hoc scoring will be performed by a rater with >5 years’ experience using TGMD for research and clinical purposes. Raw sub-scale scores and total scores will be analyzed as has been done in other studies (Allen et al., 2017; Webster et al., 2019), particularly because the current normative data is based on North American populations.

It is noted that TGMD-3 tests thirteen gross motor skills (i.e., six locomotor and seven object control skills), while the training program targets three skills each among the locomotor, object control, and stability components of fundamental movement skills; these were found to be consistent with the local curriculum context. Because developmental progressions are typically found between skills and between components of fundamental movement skills (Donnelly et al., 2017), we expect that TGMD-3 will be able to capture consequent changes following the training program.

#### Outcome: Executive Function and Working Memory

Executive function has been proposed to have two core components: working memory and inhibitory control (Diamond, 2016). These two components, however, are relatively undifferentiated in children aged three to five years, such that they fall along a single factor and have been conceptualized as a unitary construct (Nelson et al., 2017). As such, we will measure executive function using the Head-Toes-Knees Shoulders (HTKS), which is a validated task that taps both working memory and inhibitory control (Cameron Ponitz et al., 2008, 2009; McClelland et al., 2014) and is compatible with the undifferentiated nature of executive functioning in early childhood (Garon et al., 2008). The HTKS had been found to have excellent inter-rater reliability and internal consistency in studies involving young children in Hong Kong (e.g., Chung, 2015; Chung et al., 2017; Liu et al., 2018).

The HTKS is administered as a two-part game in which children perform the opposite action to verbal commands. The first part of the game involves two commands (i.e., “touch your head” and “touch your toes”) and the second part of the game involves four commands (“touch your head,” “touch your toes,” “touch your knees,” and “touch your shoulders”). When a verbal command is given (e.g., “touch your toes”), the correct response by the child is the opposite (e.g., child touches their head); the children are asked to respond to the commands as fast as possible. There are four practice trials in each part of the game, during which the examiner demonstrates the correct response to each verbal command. There are 20 test trials, which are scored as 0 (incorrect action), 1 (initially incorrect, but self-corrected and finished the correct action), or 2 (correct action). As such, the total score ranges from 0 to 40. A ceiling rule will be applied, in which a child must earn at least four points in part one in order to progress to part two (Liu et al., 2018).

As the advantages of the error-reduced learning approach are believed to be associated with its relative lack of dependence on working memory (Maxwell et al., 2001), visuospatial and verbal working memory will also be measured. Visuospatial working memory will be measured using the backward Corsi block task (Pagulayan et al., 2006), and verbal working memory will be measured using the backward digit recall test (Alloway and Archibald, 2008). Backward tasks measure working memory because they require the storage of information while additional cognitive processing is required by reversing the sequence. Both of these tests have been used in studies of children (e.g., Bull et al., 2008; Gade et al., 2017; Capio et al., 2018). In the Corsi block task, the examiner taps on a sequence of cubes, while the child watches; the child will then be asked to tap the cubes in reverse sequence. The length of the sequence will start at two items, increasing by one cube after two trials at each length until the child fails to correctly reproduce the reversed sequence on two trials of the same length. In the digit recall test, the examiner will read a sequence of numbers starting from two items, and the child will be asked to recite the digit sequence in reverse. The length of the number sequence is increased by one item after two trials at each length, until the participant fails to recite the reversed sequence correctly on the two trials of the same length. A score of 1 will be given for every trial in which the child correctly taps or recites the sequence in reverse; the sum of scores represents the visuospatial and verbal working memory scores, respectively. The longest sequence that the participant tapped or recited in reverse represents the visuospatial and verbal working memory spans, respectively.

#### Outcome: Social Competence

Teachers will be asked to rate each child’s social competence in the classroom using the Social Competence and Behavior Evaluation Short Form (SCBE-30; LaFreniere and Dumas, 1996). The SCBE-30 is a validated measure of children’s affect modulation capacity, which includes 30 items that are rated on a 6-point Likert-type scale (1 is never and 6 is always). The items make up three subscales: anger-aggression (e.g., easily frustrated), anxiety-withdrawal (e.g., uneasy in a group), and social competence (e.g., takes pleasure in own accomplishment). The minimum raw score is 10, and the maximum score is 60 in each subscale. The SCBE-30 has been used in studies involving teachers of young children in Hong Kong and has been found to have good reliability and internal consistency (e.g., Chiu and Lau, 2018; Lam and Wong, 2018).

#### Covariates

Baseline measurements of height (cm) and body mass (kg) will be taken using standard portable stadiometer (SECA 213, SECA GmbH & Co. KG, Hamburg, Germany) and weighing scale (Tanita BF679W, Tanita Corporation, Tokyo, Japan), respectively, to calculate body composition. Fine motor skills proficiency will be assessed at baseline and at follow-up as a covariate because it has been shown to mediate the correlation between gross motor skills and cognitive development (Davis et al., 2011). A systematic review has also shown that fine motor skills are associated with working memory (van der Fels et al., 2015). The fine motor sub-scale of the Hong Kong Early Child Development Scale (Rao et al., 2013) will be used. Physical activity and sedentary behavior of participants will also be assessed at baseline and at follow-up as a covariate because these have been shown to be associated with motor proficiency of children (Logan et al., 2014). Further, Pesce et al. (2016) identified physical activity outside of school as a factor that mediates the effect of movement skills training on cognitive functions. Selected items from the parent-proxy Children’s Physical Activity Questionnaire (CPAQ; Corder et al., 2009) will be used, which include items that allow parents to report the amount of time that their child spent in moderate to vigorous intensity physical activities outside of school. CPAQ is being used in an ongoing study of child development in Hong Kong following standard procedures of translation and back-translation (Brislin, 1970); it has been found to have good internal consistency (Cronbach’s alpha = 0.762).

### Data Analysis

A two-group growth model for intervention studies (Muthen and Curran, 1997) will be fitted to the outcomes data. Growth curves will be fitted to the repeated measures with separate random intercepts and slopes estimated for the training and active control groups. The random intercepts capture individual differences in skills at commencement. The intercepts will be regressed on participant characteristics (i.e., sex, age, body composition, fine motor skills, and physical activity), with the latter acting as explanatory variables for the observed individual differences. A separate but parallel model will be estimated with the intercepts set at the final follow-up to examine the extent to which the same characteristics explain differences at that final data point.

Training effects will be evaluated using random slopes fitted to the data from the training and active control groups. For both groups, separate slopes will be fitted to describe (a) the normative growth observed and (b) growth that deviates from the normative pattern that can be attributed to training. Whether the mean of this second curve deviates significantly from zero serves as a test for the effects of training. Shared variance attributable to children belonging to the same class will be corrected for potential clustering effects by using the complex sampling procedure in Mplus 8.0 (Muthén and Muthén, 2017).

## Discussion

Moving efficiently, and having the self-confidence associated with it, underpins the ability of people to explore and interact with their environment throughout their life course. Interactions with the world and other people are enabled by movement proficiency in early childhood and are believed to stimulate cognitive and social development. Investing in the motor development during early childhood, therefore, stands to generate significant benefits. However, the knowledge base needed to support such critical investment – and therefore achieve the potential positive impact – needs to be clarified through robust empirical evidence.

This present study aims to contribute to achieving the proposed potential impact by building on our knowledge and understanding of the relationships between motor, cognitive, and social development in early childhood. It is widely understood that motor development is interconnected with other domains of child development, as shown by associations revealed by cross-sectional and longitudinal studies (e.g., Bart et al., 2007; Veldman et al., 2019). Causal mechanisms are much less understood – is enabling a young child to move proficiently a necessary antecedent to effective thinking and interaction with the world? A clearer understanding of causal relationships will provide robust foundations for researchers and practitioners to collaboratively design holistic early childhood programs. This study will also evaluate potential intervening factors that need to be considered and addressed when promoting movement programs in early childhood education and care contexts. The fact that the intervention is situated in a local kindergarten strengthens our subsequent ability to translate the research findings to early childhood education policy and practice. Specifically, we may be able to roll out the training program more widely to other kindergartens and should meaningful benefits be found. However, it also needs to be acknowledged that the situational context could potentially weaken the intervention fidelity because several teachers will be involved in delivering the intervention. To mitigate this potential weakness, the research team will ensure adequate implementation monitoring and fidelity check.

We acknowledge that using TGMD-3, as a measure of gross motor skills (i.e., locomotor and object control) does not adequately reflect the three components of fundamental movement skills training that will be implemented. Stability is a component of fundamental movement skills (Donnelly et al., 2017) and is being targeted in the co-designed training program. A validated test battery for stability skills had been considered, but this test applies for children aged six to ten years (Rudd et al., 2015). Because testing the psychometric properties of such test battery for children aged three to five years is outside the scope and resources of this current study, this particular outcome will be limited to gross motor skills proficiency. In follow-up work, tests of stability skills for young children should be explored. Lastly, we also acknowledge that verbal and non-verbal intelligence could be a relevant covariate in the expected cognitive outcomes. Intelligence and working memory have been found to be highly correlated (Kyllonen and Christal, 1990) but are still distinguishable (Conway et al., 2003). However, we considered that measures of working memory have been shown to be more predictive of academic proficiency than are measures of intelligence (e.g., Lee et al., 2009). Therefore, we would keep our focus on the unitary construct of executive function (measured by HTKS), and the verbal and visuospatial components of working memory.

We propose that the potential impact of this study lies in enabling early childhood educators to effectively support children to move confidently, interact with their environments, and adopt behaviors that contribute to wellbeing that tracks into adulthood.

## Ethics and Dissemination

The study had been registered with, and is publicly available through, the Open Science Framework (OSF) Registries (registration DOI 10.17605/OSF.IO/K9DW8). The training program will be delivered in the context of kindergarten classes. As such, principals will first provide written consent for their school participation. Subsequently, parents of participants will provide written consent, and the participants will be asked for verbal assent prior to any activity (including the measurements). For participants who do not consent to participate in the study, they may join the training activities that are delivered in class, but none of the measurements will be administered for them.

The findings of this study will be disseminated in scientific conferences and peer-reviewed journals within the areas of child development and motor learning. Insights from the implementation will also be shared with the early childhood education sector to inform policy and practice. Because the study will generate further knowledge and understanding about aspects of balanced child development, the findings are expected to contribute to delivering the stipulations of the kindergarten curriculum guidelines of Hong Kong (Curriculum Development Council, 2017) and to subsequent review of the said guidelines. Knowledge will also be shared with teachers and parents to support integrated efforts at promoting motor, cognitive, and social development of children in the early years.

## Ethics Statement

The study was reviewed and approved by the Human Research Ethics Committee of the Education University of Hong Kong (reference number 2018-2019-0180). Written informed consent to participate in this study will be provided by the participants’ legal guardian/next of kin.

## Author Contributions

CC is the principal investigator of the grant and is responsible for the overall concept and design of the study. KL, RJ, and RM are named co-investigators in the grant and collaborated during the grant preparation. CC drafted the manuscript, and all co-investigators provided expert input within their areas of expertise. All authors contributed to this paper and approved the submission.

### Conflict of Interest

The authors declare that the research was conducted in the absence of any commercial or financial relationships that could be construed as a potential conflict of interest.
